# Evaluation of ChatGPT Responses About Sexual Activity After Total Hip Arthroplasty: A Comparative Study with Observers of Different Experience Levels

**DOI:** 10.3390/jcm14092942

**Published:** 2025-04-24

**Authors:** Batuhan Gencer, Ufuk Arzu, Serdar Satılmış Orhan, Turgut Dinçal, Mehmet Ekinci

**Affiliations:** Department of Orthopedics and Traumatology, Marmara University Pendik Training and Research Hospital, 34890 Istanbul, Turkey; drufukarzu@gmail.com (U.A.); serdarorhan@gmail.com (S.S.O.); tdincal@yahoo.com (T.D.); dr.ekincimehmet@gmail.com (M.E.)

**Keywords:** ChatGPT, sexual activity, total hip arthroplasty, artificial intelligence, patient education

## Abstract

**Background/Objectives**: Despite the rising tendency to depend on ChatGPT for medical counselling, it is imperative to evaluate ChatGPT’s capacity to address sensitive subjects that patients often hesitate to discuss with their physicians. The objective of this study was to evaluate the recommendations provided by ChatGPT for sexual activity subsequent to total hip arthroplasty (THA) by orthopaedic surgeons with varying degrees of experience, as well as using standardized scoring systems. **Methods**: Four patient scenarios were developed, reflecting different ages and indications for THA. Twenty-four questions were asked to ChatGPT 4.0, and responses were evaluated by three different orthopaedic surgeons. All responses were also scored using defined standardized scales. **Results**: No response was found to be ‘faulty’ or ‘partial’ by any of the observers. While the lowest mean score was attributed by the orthopaedic surgeon with less than five years of experience, the highest mean score was attributed by the orthopaedic surgeon with more than 15 years of experience but not actively working in the field of arthroplasty. An analysis of the data across scenarios revealed that in general, the scores decreased in the more specialized scenarios (*p* > 0.05). **Conclusions**: ChatGPT shows potential as a supplementary resource for addressing sensitive postoperative questions such as sexual activity after THA. However, its limitations in providing nuanced, patient-specific recommendations highlight the need for further refinement. While ChatGPT can support general patient education, expert clinical guidance remains essential for addressing complex or individualized concerns.

## 1. Introduction

With the rapid advancement of artificial intelligence (AI) in healthcare, large language models like ChatGPT have emerged as widely accessible and user-friendly tools for medical information retrieval [1,2,3,4,5,6,7,8,9,10,11,12,13]. ChatGPT, based on generative pre-trained transformer (GPT) architecture, is designed to generate human-like responses to diverse inquiries, including those related to medical and orthopaedic topics [1,2,3]. While AI-powered chatbots hold the potential to bridge gaps in patient education, concerns remain regarding their accuracy, consistency, and clinical reliability [4,5,6,7]. Inaccurate information or AI models’ improperly detailed reactions may cause needless worry, poor postoperative care, or even a higher risk of complications [8]. In 2023, Alowis et al. emphasized the potential of AI-enabled chatbots to facilitate clinicians in diagnosis and personalized treatment planning. Nevertheless, they emphasized challenges pertaining to data privacy, bias, and the necessity for human expertise [14].

Total hip arthroplasty (THA), otherwise known as the replacement of the hip joint following primary or secondary coxarthrosis, is one of the most effective and widely performed orthopaedic procedures, significantly improving patient mobility, alleviating pain, and enhancing the overall quality of life [1,9,10]. In view of the global demographic shift towards an ageing population, there has been considerable growth and innovation in the field of joint replacement surgery. Indeed, primary hip and knee arthroplasty is reported to be among the top five most frequently performed and fastest growing procedures among all surgical disciplines in the United States [15,16]. In addition to the evolution of surgical methodologies, minimally invasive techniques, and the development of implant technologies, the postoperative rehabilitation process is another pivotal element in determining patient-reported outcomes and satisfaction following total hip arthroplasty. Despite these advantages, some elements of postoperative recovery are still not well covered in standard clinical conversations. One such area is sexual activity, a crucial but sometimes neglected aspect of wellbeing [11,12]. Research indicates that many patients experience anxiety and uncertainty regarding the safety, timing, and recommended positions for resuming sexual activity after THA [9,13]. However, these issues are often ignored due to disgrace, perceived stigma, or a lack of structured guidance from medical professionals [8]. Based on our clinical findings, we know that patients are often hesitant to ask orthopaedic surgeons about this topic before or after surgery, especially in traditional societies like ours. This phenomenon is particularly pronounced in cases where the patient is female and the surgeon is male. Unfortunately, the frequency of total hip arthroplasty is higher in women, and there is a male predominance among orthopaedic surgeons [17,18,19].

This study aims to evaluate ChatGPT’s recommendations to questions regarding sexual activity subsequent to THA by orthopaedic surgeons with varying degrees of experience, as well as using standardized scoring systems. By systematically assessing the accuracy, comprehensiveness, and consistency of AI-generated responses, this research seeks to determine the feasibility of integrating AI tools into patient education while acknowledging their limitations and emphasizing the continued necessity of expert clinical judgment. Understanding both the capabilities and limitations of ChatGPT in addressing sensitive orthopaedic topics is essential for ensuring safe and effective patient guidance, given the growing reliance on AI-driven medical information.

## 2. Materials and Methods

This study did not use patient data, and the assessment of artificial intelligence support was conducted through author-generated patient scenarios; hence, obtaining ethical committee or institutional review board approval was considered unnecessary, in accordance with similar studies in the literature [4,6,9,20]. Artificial intelligence was used exclusively in answering the questions of the scenarios created in the form of AI-powered chatbots. It is important to note that the present study did not make use of artificial intelligence in any other aspect, such as data analysis, interpretation, or the composition of the manuscript.

In determining the patient scenarios, the existing literature on hip arthroplasty was thoroughly reviewed, and four patient scenarios with distinct characteristics were meticulously designed in English: a 65-year-old male with primary coxarthrosis who underwent THA, a 40-year-old female with dysplastic coxarthrosis and symptomatic leg-length discrepancy who underwent subtrochanteric shortening femoral osteotomy combined with THA, a 40-year-old female with bilateral dysplastic coxarthrosis without any leg-length discrepancy who underwent bilateral THA in the same session, and a 75-year-old male who underwent THA after an osteoporotic femoral neck fracture he experienced two days ago (Appendix A). The questions for each scenario were then selected by reviewing the current literature on sexual activity following THA [21,22,23]. The questions centred on assessing the capacity of artificial intelligence to provide information to patients, its capacity to offer appropriate guidance, its proficiency in accurately identifying and describing appropriate sexual positions, and the ability to clarify any potential risks. The questions were prepared as open-ended by two board-certified orthopaedic specialists who are actively involved in patient care in orthopaedics, perform arthroplasty surgeries, have previously participated in survey studies, and possess native English proficiency. A total of 24 questions were asked to ChatGPT (ChatGPT Version 4.0; OpenAI, 2024), with the same six questions in each patient scenario. Questions were asked to ChatGPT 4.0 in an ordered manner, similar to how patients often ask questions. This approach involved first defining the characteristics specified in the scenario and then indicating that the patients were on the first postoperative day (i.e., “Hello ChatGPT, I’m a 65-year-old guy, and I had hip replacement surgery yesterday because of hip arthritis. Can I ask you some questions about sexual activity after the surgery?”) (Appendix A). It should be noted that no question was repeated in the same scenario, a methodological decision made to reflect the typical user experience and assess ChatGPT’s immediate response performance.

All answers given by ChatGPT 4.0 were recorded and then analysed. First, all responses were scored by three different orthopaedic surgeons with varying degrees of experience and without being aware of each other’s responses. The first observer (Observer No. 1) was an orthopaedic surgeon with less than five years of experience; the second observer (Observer No. 2) was an orthopaedic surgeon with more than 15 years of experience but not actively working in the field of arthroplasty (<50 operations per year); and the third observer (Observer No. 3) was an orthopaedic surgeon with more than 15 years of experience and actively working in the field of arthroplasty (>200 operations per year). All three observers were tasked with evaluating the responses generated by ChatGPT 4.0, focusing on assessing the accuracy, comprehensiveness, and consistency of the responses. The observers were instructed to rate the responses on a scale of 1 to 5. A comprehensive description of the used Likert scale is provided in Table 1. Further, the same observers were asked to reassess the responses three months after their initial evaluation, and intra-rater reliability was evaluated. Considering ChatGPT’s self-improving and learning algorithm, each answer given after the first inquiry was recorded and re-evaluated three months later. The decision to avoid re-asking the questions to ChatGPT ensured the preservation of intra-observer reliability, given the potential for the artificial intelligence algorithm to undergo self-improvement during the intervening period.

Following the scoring by the observers, the responses generated by ChatGPT 4.0 were also evaluated using standardized scoring systems, assessing the quality of the information shared by the model: The Modified DISCERN (mDISCERN) Score, and the Journal of the American Medical Association (JAMA) Benchmark Criteria [24,25,26]. The mDISCERN score is a 5-point assessment questionnaire derived from the 16-question DISCERN Health Information Evaluation Score [24,26]. This 5-point score was calculated for each patient scenario, earning 1 point for each “Yes” and 0 points for each “No”, and higher scores were considered indicative of improved reliability (Table 2). The JAMA Benchmark Criteria constitutes an evaluation scale comprising four recognized criteria (authorship, attribution, disclosure, and currency) that assess the quality of information. In this scoring system, in which each criterion is evaluated with a value of 1 point, and again higher scores were interpreted as improved reliability, the authorship criterion refers to the disclosure of the author’s identity, the attribution criterion refers to the appropriate sharing of citations and copyright information, disclosure criterion refers to the emphasis of potential conflicts of interest, and the currency criterion refers to the date of the information provided [25,26] (Table 2). The evaluation of both scoring systems for each patient scenario was performed twice by an independent observer (observer No. 4) with a three-month waiting period in between. The second evaluation was performed using the archived answers from the first questioning, rather than re-asking the same questions to ChatGPT.

Statistical analyses were conducted using IBM^®^ SPSS^®^ Statistics Version 26. Descriptive statistics were presented as mean, standard deviation, and minimum–maximum range values. Cohen’s Kappa analysis was used for the intra-rater reliability analysis. The agreement level analysis of Kappa values was interpreted as follows: 0–0.2 was interpreted as ‘none’, 0.21–0.39 as ‘minimal’, 0.40–0.59 as ‘weak’, 0.60–0.79 as ‘moderate’, 0.80–0.90 as ‘strong’, >0.90 as ‘almost perfect’, and 1.00 as ‘perfect’ agreement. The Kruskal–Wallis test was employed to compare the ratings provided by observers across different scenarios. For this study, *p*-values less than 0.05 were considered statistically significant.

## 3. Results

### 3.1. Observer Assessments

All observers conducted their assessments on two separate occasions at three-month intervals, and an ‘almost perfect’-to-‘perfect’ agreement was found between the observations (Table 3).

**Figure 1:**
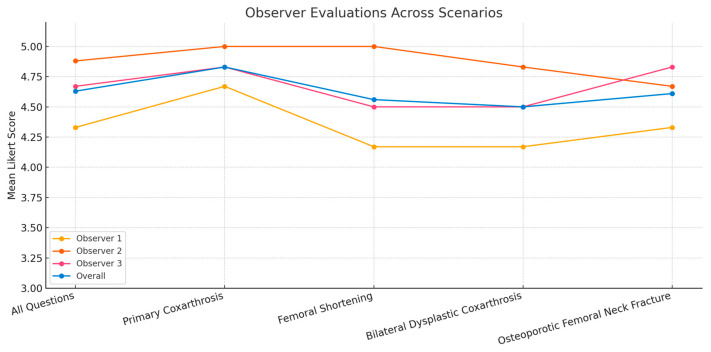
Observer evaluations across scenarios.

Among the responses provided by ChatGPT 4.0, no response was found to be ‘faulty’ or ‘partial’ by any observers. The mean score of the responses was 4.63 ± 0.615 (Range: 3–5). While the lowest mean score was attributed by Observer No. 1 (orthopaedic surgeon with less than five years of experience), the highest mean score was attributed by Observer No. 2 (orthopaedic surgeon with more than 15 years of experience but not actively working in the field of arthroplasty) (Figure 1). A thorough examination of the data revealed that the highest mean score for ChatGPT responses was attained in the first scenario (primary coxarthrosis), with diminished mean scores observed in the other scenarios (dysplastic coxarthrosis and osteoporotic fracture). However, these differences were not statistically significant (*p* > 0.5) (Table 4).

### 3.2. Standardized Assessments

In the context of evaluating the responses through the standardized scoring systems, a scenario-based analysis was conducted, and the responses for each scenario were then subjected to evaluation. The average mDISCERN score was 2.25 ± 0.5 out of 5, whereas the average JAMA Benchmark score was 1 ± 0 out of 5. The areas where points were lost were due to the lack of source material and failure to share additional sources of information, as well as the failure to emphasize areas of uncertainty in specific scenarios. It was observed that the JAMA Benchmark score was calculated as ‘1’ point for all scenarios due to ChatGPT not sharing author, citation, and disclosure information (Table 5).

## 4. Discussion

The advent of the internet, the subsequent popularity of social media, and the current accessibility of artificial intelligence have collectively precipitated a paradigm shift within the healthcare industry [27,28,29,30]. AI-driven healthcare technologies, such as chatbots, rapidly transform the healthcare system by offering viable alternatives to traditional patient care methods [28,29]. In addition to their numerous recommended applications, one of the most notable features of chatbots is their capacity to respond to queries that patients are hesitant to ask to their physicians [1,2,3,4,6,8,9,28,29]. This is particularly notable in some societies, where patients may be reluctant to discuss delicate topics with their physicians, such as postoperative sexual activity. One of the most critical aspects of this study is the evaluation of ChatGPT’s responses by observers with varying levels of expertise. This approach unveils the observer level to which the chatbot’s answers most closely align. The most important finding of the study was that the responses provided by ChatGPT 4.0 were ranked the highest score by the orthopaedic surgeon with over 15 years of experience, though not currently working in the field of arthroplasty, whereas the lowest score was ranked by the orthopaedic surgeon with less than five years of experience. Furthermore, we observed that ChatGPT 4.0 provides standardized, evidence-based responses but had difficulty generating personalized recommendations for patients in specific situations (like dysplastic coxarthrosis or osteoporotic fractures), and the accuracy and comprehensiveness of its responses show a small decline.

The findings of this study indicate that ChatGPT 4.0 has the potential to function as a supplementary educational tool for patients undergoing THA regarding postoperative sexual activity, though it is not a standalone solution, considering that ChatGPT 4.0 did not give any “faulty” or “partial” answers, gave at least “fair” answers, and had satisfactory scores (4.63 ± 0.615, range: 3–5). Similarly, in 2024, Hu et al. asked ChatGPT 12 inquiries concerning periprosthetic joint infections and noted that the responses did not include any ‘unsatisfactory’ responses [4]. Magruder et al. utilized a Likert scale ranging from 1 to 5 in their study, in which they analysed the responses of ChatGPT to questions pertaining to total knee arthroplasty, and they observed that ChatGPT scored above average (>3 points for each) [20]. Conversely, Wright et al. asked 30 questions related to total knee and hip arthroplasties to ChatGPT and evaluated the responses, finding that only 59.2% of the answers were deemed satisfactory [9]. Mika et al. asked ten frequently asked questions concerning THA to ChatGPT, subsequently reporting that the chatbot’s responses were found to be unbiased and evidence-based, even on controversial issues. It was further noted that ChatGPT provided an ‘unsatisfactory’ response in only one instance [30]. The existing literature seems to lack agreement about the influence of ChatGPT on patient education, but there is a consensus that the responses are evidence-based and objective. It is remarkable that while the aforementioned studies addressed common inquiries, our investigation focused on a more specific subject. The predominance of ‘adequate’ and ‘excellent’ responses, with a complete absence of ‘faulty’ or ‘partial’ answers, underscores the potential for ChatGPT 4.0 to be employed in specific domains, such as sexual activity after THA, with clinician supervision.

In the context of an observer-based analysis, it is noteworthy that the scores assigned by the first observer are the lowest (4.33 ± 0.761), while those assigned by the second observer are the highest (4.88 ± 0.338). As mentioned before, the first observer is an orthopaedic surgeon with less than five years of experience; thus, he is younger than his colleagues. The most significant difference between him and the other observers, after experience, is his age. As a matter of fact, when he was asked what was missing in the questions he rated as ‘fair’, he highlighted his expectation of more from artificial intelligence. He further elaborated that, in his opinion, an interface with such extensive potential should leverage visual elements to inform the patient, yet he observed no visual components in any of the responses. In contrast, the other two observers did not anticipate a visual response from ChatGPT. That is to say, age may be a significant factor in expectations of artificial intelligence. It should be noted that the ages of the second and third observers are relatively close to each other, and the most notable difference between these two observers is their experience in arthroplasty. The third observer, who is actively engaged in arthroplasty, can more clearly evaluate the movement vectors of the hip during sexual activity, and he also takes into account the complications he encountered. Consequently, he found the chatbot’s answers more inadequate on several points. Conversely, the second observer, who may overlook these minor technical nuances, deems the responses provided by ChatGPT 4.0 to be satisfactory, as the second observer attains the highest mean score (4.88 ± 0.338). It is imperative to emphasize that the present study exclusively included orthopaedic physicians as observers. While sexual activity is multidisciplinary in nature, as was stated in the study, the aim was to compare the responses from AI with possible responses of the physicians, with whom patients had a serious dialog before surgery. To this end, the responses were evaluated using a sample of physicians with varying levels of experience and ages; hence the exclusion of other healthcare professionals such as nurses or rehabilitation specialists.

A subsequent analysis of the standardized scoring systems reveals that ChatGPT 4.0 received very low scores, which can be attributed to the absence of author, reference, and disclosure information. Furthermore, when ChatGPT is prompted to provide references, the chatbot demonstrates a substantial foundation of literature, though there may be instances of repetition and unsuitable citations [31]. The results of analysing artificial intelligence with standardized systems have been shown to be similar in the literature. In 2024, Kasapovic et al. utilized the mDISCERN score in their study evaluating the use of ChatGPT in orthopaedics and traumatology and reported an average score of 2.4 [32]. In a subsequent study, Hancı et al. reported an average mDISCERN score of 2.29 for ChatGPT responses related to palliative care [33]. In the same year, Gül et al. reported that 89% of ChatGPT responses had an mDISCERN score of 2 out of 5 in their study investigating responses related to subdural hematoma [34]. In 2023, Ulusoy et al. evaluated the quality-of-life-related responses of ChatGPT and calculated the JAMA Benchmark score as 0 out of 4 [35]. In 2024, Reyhan et al. evaluated the keratoconus-related information of ChatGPT and calculated the average JAMA Benchmark score as 1.4 out of 4 [36]. We hypothesized that the low scores given to ChatGPT are attributable to the fact that these scoring systems were developed to verify the accuracy of information shared on the internet and social media rather than artificial intelligence. In the future, alternative scoring systems will need to be developed to more objectively reveal the effects of artificial intelligence on medical information sharing and patient education. Conversely, the absence of author, reference, and disclosure information, which is exhibited among the rationales for these lower standardized scores, constitutes a significant usage constraint of AI-based chatbots.

In the course of a scenario-based evaluation, it was observed that patients exhibiting specific characteristics received lower scores. In fact, despite the changing scenarios, the majority of the responses from ChatGPT 4.0 remained consistent, a finding that was noted by all observers. This suggests that ChatGPT 4.0 accesses standardized and evidence-based information but lacks the capacity to provide patient-specific recommendations for patients exhibiting traits. This emphasizes the necessity for clinician oversight in combination with AI-based chatbots, particularly in tailored patient management.

Beyond accessibility and consistency, ChatGPT’s ability to provide immediate responses allows patients to seek guidance at any time without the need for scheduling consultations [1]. This can reduce anxiety in patients who may feel uncomfortable discussing sexual health directly with their healthcare providers, especially in traditional societies like ours. However, ChatGPT’s responses, though comprehensive, lack the ability to adjust recommendations based on real-time patient assessments, medical history, or nuanced recovery progress, which are crucial in postoperative care. This underscores the necessity of integrating AI tools with direct clinician oversight to ensure tailored, contextually appropriate recommendations. Collaborative efforts between AI developers, medical professionals, and researchers will be essential to refine ChatGPT’s application in orthopaedic rehabilitation and optimize its role in patient education.

This study is distinguished by its evaluation of ChatGPT’s proficiency, accuracy, comprehensiveness, and consistency in specific subjects. While the literature typically focuses on the proficiency and accuracy of chatbots’ responses to frequently asked questions within a particular domain [4,7,9,17,30,31,33,34,36], this study is noteworthy for its pioneering investigation into the competence of artificial intelligence in such a highly specialized and sensitive field (sexual activity following THA) while also highlighting the necessity of clinician supervision. Another significant aspect of the study is the evaluation of ChatGPT’s responses by observers with varying levels of expertise, which adds a valuable perspective to the research.

This study is not without its limitations. Despite the existence of numerous reasons for patients to be reluctant to discuss sexual matters with their physician following THA, such as disgrace, perceived stigma, or a lack of structured guidance from medical professionals, the most significant underlying cause of this reluctance is the low social status of patients. It is uncertain whether patients with a low social status can adequately master and utilize AI-based programs. Furthermore, the study is unable to assess a patient–chatbot dialogue, which may facilitate the generation of follow-up questions. It is essential to note that patients receiving information about sexual activity after THA are likely to seek clarification through repetitive questions to ChatGPT when they encounter uncertainties or have unanswered queries. However, it is not possible to evaluate the possible answers to those questions. Finally, it is vital to acknowledge that our patient scenarios, although prepared diligently, do not replace data obtained directly from the patients. It is also imperative to emphasize that the study’s primary focus was on the evaluation of responses from ChatGPT 4.0, and it is acknowledged that the outcomes may vary when applied to other AI-based chatbots.

## 5. Conclusions

ChatGPT 4.0 demonstrated the ability to provide clear and general guidance about sexual activity after THA, with a significant proportion of its answers rated as “adequate” or “excellent”. However, the limitations of ChatGPT include its inability to anticipate patient-specific risk factors, technical details, and clinical nuances, as well as its inability to meet the high expectations of younger surgeons.

In conclusion, while ChatGPT 4.0 can support general patient education and shows potential as a complementary resource for addressing sensitive postoperative issues such as sexual activity after THA, expert clinical guidance remains essential to address complex or individualized concerns. In the future, a combined approach, utilizing AI for general education and surgeons for personalized advice, may enhance patient outcomes.

## Figures and Tables

**Table 1:** Likert Scale developed to assess the accuracy, comprehensiveness, and consistency of the answers from ChatGPT 4.0.

Scale	Definition	
**1**	**Faulty**	The language bot’s answers are factually inaccurate and misleading.
**2**	**Partial**	It is clear that important gaps in the data exist, despite the fact that some information on the subject has been shared.
**3**	**Fair**	It covers the main points, but it could be better.
**4**	**Adequate**	It is comprehensive and detailed. Only minor details/technical points have been overlooked.
**5**	**Excellent**	It is highly detailed and comprehensive. It covers all aspects of the problem and provides a deep understanding.

**Table 2:** Scoring systems assessing the quality of the information shared by ChatGPT.

Modified DISCERN Score	
Question	Definition
**1**	Are the aims clear and achieved?
**2**	Are reliable sources of information used?
**3**	Is the information presented both balanced and unbiased?
**4**	Are additional sources of information listed for patient reference?
**5**	Are areas of uncertainty mentioned?
**Journal of the American Medical Association (JAMA) Benchmark Criteria**	
**Question**	**Definition**
**1**	**Authorship**—Disclosure of the author’s identity
**2**	**Attribution**—Appropriate sharing of citations and copyright information
**3**	**Disclosure**—The emphasis on potential conflicts of interest
**4**	**Currency**—The date of the information provided

**Table 3:** Analysis of intra-rater reliability.

	Intra-Rater Reliability	
	Kappa	Level of Agreement
Observer No. 1	0.931	Almost perfect
Observer No. 2	1.000	Perfect
Observer No. 3	0.908	Almost perfect
Standardized Scoring Systems	1.000	Perfect

**Table 4:** Evaluation of ChatGPT responses by observers of different experience levels.

	Observer No. 1	Observer No. 2	Observer No. 3	Overall Scores
**All Questions** (*n* = 24)	4.33 ± 0.761 (3–5)	4.88 ± 0.338 (4–5)	4.67 ± 0.565 (3–5)	4.63 ± 0.615 (3–5)
**First Scenario** (*n* = 6)	4.67 ± 0.516 (4–5)	5 (5)	4.83 ± 0.408 (4–5)	4.83 ± 0.383 (4–5)
**Second Scenario** (*n* = 6)	4.17 ± 0.983 (3–5)	5 (5)	4.5 ± 0.548 (4–5)	4.56 ± 0.705 (3–5)
**Third Scenario** (*n* = 6)	4.17 ± 0.753 (3–5)	4.83 ± 0.408 (4–5)	4.5 ± 0.837 (3–5)	4.5 ± 0.707 (3–5)
**Fourth Scenario** (*n* = 6)	4.33 ± 0.816 (3–5)	4.67 ± 0.516 (4–5)	4.83 ± 0.408 (4–5)	4.61 ± 0.608 (3–5)
***p***	0.667	0.260	0.556	0.444

N: number of questions, *p*: statistical significance value. Mean ± standard deviation (minimum–maximum range) values were used as descriptive statistics.

**Table 5:** Evaluation of ChatGPT responses by standardized scoring systems.

	Modified DISCERN Score	JAMA Benchmark Score
**Answers from the First Scenario**	3	1
**Answers from the Second Scenario**	2	1
**Answers from the Third Scenario**	2	1
**Answers from the Fourth Scenario**	2	1
**Overall**	2.25 ± 0.5 (2–3)	1 ± 0 (0–1)

Mean ± standard deviation (minimum–maximum range) values were used as descriptive statistics.

## Data Availability

The datasets generated during and/or analysed during the current study are not publicly available, but are available from the corresponding author on reasonable request.

## Abbreviations

The following abbreviations are used in this manuscript:

AI	Artificial Intelligence
THA	Total Hip Arthroplasty
JAMA	Journal of the American Medical Association

## Appendix A

### Appendix A.1. Scenarios and Questions

**SCENARIOS**
**S1:** Hello, ChatGPT. I’m a 65-year-old guy, and I had hip replacement surgery yesterday because of hip arthritis. Can I ask you some questions about sexual activity after the surgery?
**S2:** Hi, ChatGPT. I’m a 40-year-old woman. I had one-sided developmental hip dysplasia as a child. This resulted in arthrosis and shortness in my hip, and I had hip replacement surgery yesterday. My doctor warned me not to step on it, but since I’m reluctant to ask him, can I ask you a few questions about my sexual activity after the surgery?
**S3:** Hi ChatGPT, I’m a 40-year-old woman. I’ve had early arthritis in both hips because of developmental hip dysplasia since I was a kid. My legs were at the same length, but yesterday, I had prosthetic surgery on both hips at the same session because of unbearable pain. Can you answer the questions below? I was too embarrassed to ask my doctor.
**S4:** Hello ChatGPT. I had total hip replacement surgery yesterday due to a hip fracture I suffered two days ago after a fall at home. I’m a 75-year-old man, and I’d like to have some information about sexual activity after surgery. Can you answer the following questions?
**QUESTIONS**
**Q1.** When is it safe to resume sexual activity/coitus following surgery? How can I determine my readiness to resume sexual activity/coitus post-surgery? Should I wait until my pain is fully resolved for sexual activity/coitus? Should I wait for the stitches to be removed?
**Q2.** Will I experience discomfort during sexual activity/coitus?
**Q3.** What should I be aware of during sexual activity/coitus?
**Q4.** What information should I provide to my partner prior to sexual activity/coitus? What should I advise him/her about?
**Q5.** What are the safest positions for sexual activity/coitus?
**Q6.** What are the potential consequences of sexual activity/coitus? Which health issues could arise? When should I consult medical professionals?
